# Easy353: A Tool to Get Angiosperms353 Genes for Phylogenomic Research

**DOI:** 10.1093/molbev/msac261

**Published:** 2022-12-02

**Authors:** Zhen Zhang, Pulin Xie, Yongling Guo, Wenbin Zhou, Enyan Liu, Yan Yu

**Affiliations:** Key Laboratory of Bio-Resources and Eco-Environment of Ministry of Education, College of Life Sciences, Sichuan University, Chengdu, Sichuan, 610065, P. R. China; Key Laboratory of Bio-Resources and Eco-Environment of Ministry of Education, College of Life Sciences, Sichuan University, Chengdu, Sichuan, 610065, P. R. China; Key Laboratory of Bio-Resources and Eco-Environment of Ministry of Education, College of Life Sciences, Sichuan University, Chengdu, Sichuan, 610065, P. R. China; Department of Biology, University of North Carolina at Chapel Hill, Chapel Hill, NC 27599, USA; Key Laboratory of Bio-Resources and Eco-Environment of Ministry of Education, College of Life Sciences, Sichuan University, Chengdu, Sichuan, 610065, P. R. China; Key Laboratory of Bio-Resources and Eco-Environment of Ministry of Education, College of Life Sciences, Sichuan University, Chengdu, Sichuan, 610065, P. R. China

**Keywords:** angiosperms353, reference-guided assembler, high-throughput sequencing

## Abstract

The Angiosperms353 gene set (AGS) consists of a set of 353 universal low-copy nuclear genes that were selected by examining more than 600 angiosperm species. These genes can be used for phylogenetic studies and population genetics at multiple taxonomic scales. However, current pipelines are not able to recover Angiosperms353 genes efficiently and accurately from high-throughput sequences. Here, we developed Easy353, a reference-guided assembly tool to recover the AGS from high-throughput sequencing (HTS) data (including genome skimming, RNA-seq, and target enrichment). Easy353 is an open-source user-friendly assembler for diverse types of high-throughput data. It has a graphical user interface and a command-line interface that is compatible with all widely-used computer systems. Evaluations, based on both simulated and empirical data, suggest that Easy353 yields low rates of assembly errors.

## Introduction

The advancement of sequencing technologies has greatly facilitated phylogenetic studies. Recently, an increasing number of molecular markers have become available for studying phylogenetic relationships among plants. These data can illuminate complicated biological evolutionary histories, species origins, and species boundaries (Fazekas et al. 2009; Gray 2012; Edger et al. 2019; Veltjen et al. 2022). The Angiosperms353 gene set (AGS) is a set of universal low-copy nuclear genes, which were extracted from the 1KP (Leebens-Mack et al. 2019) transcriptomes. The AGS can be utilized at taxonomic scales representing either deep or shallow phylogenetic divergence (Antonelli et al. 2021; Baker, Dodsworth et al. 2021; Beck et al. 2021; Clarkson et al. 2021; Pérez-Escobar et al. 2021; Slimp et al. 2021). The number of articles employing the AGS has been increasing over time. For example, Google Scholar shows 11 publications in 2019, 17 publications in 2020, and 76 publications in 2021.

Compared with other molecular makers in phylogenetic genomics (e.g., plastid genes), the AGS offers numerous advantages (Baker, Dodsworth et al. 2021; Slimp et al. 2021). However, it is still a challenge to recover and assemble the AGS in an efficient and accurate way. According to the statistics of AGS data from ∼10,000 species on the Kew Tree of Life Explorer at https://treeoflife.kew.org (Baker, Bailey et al. 2021), the AGS data are mainly recovered from three types of high-throughput sources: target enrichment, transcriptome, and the annotated whole genome. Acquiring the AGS data from these sources can be challenging because the number of recovered genes can be largely influenced by the probes that are designed for target enrichment data as well as by poor initial RNA quality for transcriptome data, and by a limited number of well-annotated genomes (Marks et al. 2021). Pipelines that have been developed to obtain the AGS for phylogenetic research include HybPiper (Johnson et al. 2016), PHYLUCE (Faircloth 2015), and SECAPR (Andermann et al. 2018). However, the number of genes recovered from these pipelines is usually substantially far lower than 353, even when using correct enrichment steps and high coverage data (e.g., Zhou et al. 2021). Furthermore, error rates, concerning the recovered genes from pipelines, are often not assessed. In general, erroneous sequences can be caused by insufficient sequencing depth, assembly errors, and the presence of paralogous genes. The resulting inaccurate gene assemblies may lead to the misestimation of branch lengths, divergence times, and/or species relationships.

To address the above issues, we created the Easy353 software, a reference-guided gene assembler (e.g., see Schneeberger et al. 2011; Bao et al. 2014). Easy353 can capture the AGS with high accuracy and precision from data derived from the transcriptome, the whole genome, or even genome skimming sequencing data. In Easy353, we implemented an optimized filtering approach that is based on *k*-mers and an assembly algorithm that employs the weighted de Bruijn graph (DBG). We fully utilize existing Angiosperms353 gene sequences to assist in read filtering and read assembly. Easy353 is easy to install, user-friendly, and compatible with all widely-used operating systems (i.e., Linux, Windows, and macOS).

## Software Features and Availability

Easy353 consists of three primary modules for recovering the AGS from sequencing data. These modules are denoted: “Reference database building”, “Read filtering”, and “Read assembly”. Detailed descriptions of these modules are available in the supplementary information, Supplementary Material online.

### Reference Database Building

**Fig. 1.**
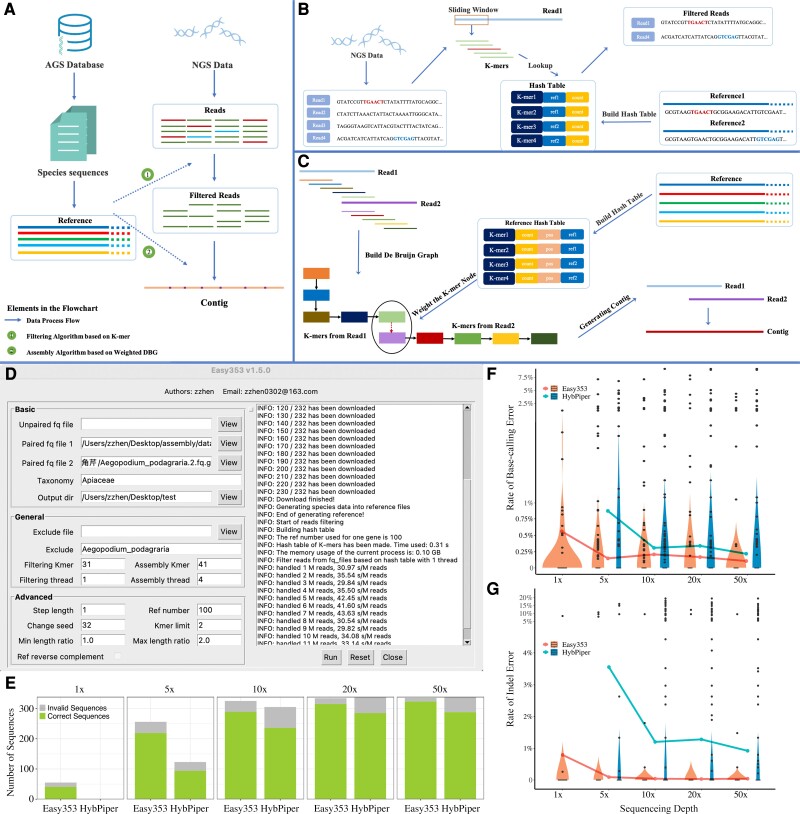
The workflow and application of Easy353. (*A*) The workflow of Easy353. Easy353 consists of three primary modules for recovering the AGS from sequencing data: reference database building, read filtering, and read assembly. First, available AGS sequences should be downloaded from Kew Tree of Life Explorer (https://treeoflife.kew.org) to make a reference database; the information on the reference sequences will be used to aid read filtering and assembly. Second, Easy353 will filter out the reads related to AGS from sequencing data. Finally, the filtered reads will be assembled into the target genes. (*B*). The workflow of read filtering. Easy353 uses the partial fragment consistency between reference sequences and reads to classify the raw reads into individual reads of gene locus. First, all reference sequences are broken into *k-*mers, which are then kept in a hash table. Then each read is processed by being divided into *k*-mers and finding out whether these *k-*mers are in the hash table to see if the read is related to the reference gene. Finally, the reads related to the same gene are stored in an individual file. (*C*). The workflow of the read assembly. Easy353 will assemble the reads related to AGS with the help of position information on reference sequences. All the reads will be divided into *k*-mers, which will be used to build the DBG. In the DBG, each node represents a *k-*mer and there will be a directed edge between two nodes if there is an overlap with *k*−1 base. Finally, Easy353 will find a path that traverses most nodes and visits each node only once as the target sequence. (*D*). The interface of Easy353-GUI. (*E–G*). Comparison of the accuracy of sequences recovered by Easy353 and HybPiper using simulated data of *Oryza sativa* with different coverage levels. (*E*). Comparison of the number of correct and invalid sequences recovered by Easy353 and HybPiper at different sequencing depths (1, 5, 10, 20, 50×). The correct sequences are those that can be utilized for phylogenetic analysis, whereas the invalid sequences have a high number of base-calling or indel errors and should not be used for phylogenetic analysis. (*F*). The base-calling error rate of sequences recovered by Easy353 and HybPiper. The violin plot shows the base-calling error rate for each recovered sequence, while the line graph shows the base-calling error rate over the total sequence length. (*G*). The indel error rate of sequences recovered by Easy353 and HybPiper. The violin plot shows the indel error rate for each recovered sequence, while the line graph shows the indel error rate over the total sequence length.

To recover the AGS from a target species, the first step of Easy353 is to build a reference database with user-defined organisms. The database could consist of the genus/family data to which the target species belongs. The available AGS sequences of the same genus/family of target species could be downloaded automatically from the Kew Tree of Life Explorer at https://treeoflife.kew.org, then the downloaded AGS sequences would be stored in FASTA format as the reference. Users could also make a customized reference database (files that contain homologous sequences of some genes in a FASTA format) by using the command line version of Easy353 (fig. 1*A*).

### Read Filtering

Although all HTS approaches can generate a significant number of reads, the reads matching the AGS are typically sparsely distributed across the genome. Filtering reads before assembly can substantially reduce assembly complexity and improve assembly accuracy. Unlike the BLASTX (Camacho et al. 2009) or BWA (Li and Durbin 2009) approaches in HybPiper, we applied a read-filtering algorithm based on *k*-mers (*k* consecutive bases in one DNA sequence). This algorithm maintains the partial fragment consistency between reference sequences and reads to classify the raw reads into different individual reads from different gene loci (fig. 1*B*).

All reference sequences were broken into *k*-mers and represented in a hash table. Then, each read was divided into *k*-mers and checked against the hash table for identical *k*-mers; if one or more identical *k*-mers were detected, this read was considered a useful read for matching to the reference gene. We modified a read-mapping algorithm based on hash tables (Schbath et al. 2012) and optimized it to quickly and accurately recover the AGS. We used long *k*-mers and improved accuracy by not allowing mismatches. Because the longest gene in the AGS is no more than 2,000 bp (Johnson et al. 2019), we accelerated computation by storing the entire reference hash table in RAM. We also utilized the *step* parameter (the length of the interval when splitting the reads into *k*-mers) to accelerate read filtering without greatly sacrificing accuracy. Finally, the reads related to the same gene were stored in the same individual file.

### Read Assembly

The essential stage was to assemble the filtered reads into different target genes. To improve the number and accuracy of the assembled genes, we developed a read-assembly algorithm inspired by previous work with weighted DBGs (Compeau et al. 2011; Cameron et al. 2017; Pandey et al. 2017) and that involves using conservative regions on reference sequences to assist assembly (fig. 1*C*).

In the DBG, each node represented a *k*-mer (independent of the *k*-mers used in read filtering), and there would be a directed edge between two nodes if overlap with *k*−1 base was detected (Liao et al. 2019). The read assembly issue was, then, turned into finding a correct path in an assembly graph by the DBG method (i.e., finding a path that traverses the most nodes and visits each node only once). To find the path, Easy353 chose the most frequent *k*-mer as the initial node and a node with *k*−1 overlaps with the initial node as the next node. When several successor nodes were identified, information on the reference sequences was used to weigh the nodes, and the subsequent node with the highest weights was selected as the next to visit. The weight of the subsequent node was calculated as *W*_*node*_ = *count*^(1−*pos*)^ , where the *count* was the abundance of the *k*-mer, and the *pos* was the distance (the average percentage distance of a *k*-mer on the reference) difference between the current *k*-mer and the next *k-*mer. Finally, the path with the most connected nodes in the DBG was selected as the assembly result. By optimizing the algorithm, Easy353's assembly method accounted for not only the most frequent bases of each site in the read but also the position information and the base frequency of each site in the reference sequences. The output of Easy353 can be further improved by our previous script PPD (Zhou et al. 2021) to identify putative paralogues.

Easy353 is an easy-to-use Python program. Due to substantial optimization, it is comparable in performance to similar C programs. In addition, Easy353 is distributed with two user interfaces: a graphical user interface (Easy353-GUI) (fig. 1*D*) and a command-line interface (Easy353). We recommend users to use the Easy353-GUI on their personal computers (either Windows or macOS without installation). Usually, Easy353 can recover the AGS of one species in <30 min, and memory utilization is typically <10 GB. Users can also reduce memory consumption by limiting the number of reference sequences.

## Software Verification

To evaluate the efficiency of Easy353 in extracting the AGS, we tested the accuracy, the number of genes, and the length of AGS genes by using both simulated and empirical data. Results were compared with those from HybPiper v2.0.1.

We first tested the accuracy of the results from Easy353. We did this by simulating data from *Oryza sativa* with gradient sequencing depths (1, 5, 10, 20, and 50×). The simulation data were produced by the ART software (Huang et al. 2011) with the CDS of *O. sativa* as reference sequences (Ouyang et al. 2006). The nucleotide FASTA format data of all CDS of *O. sativa* were obtained from Phytozome (Goodstein et al. 2011). The Angiosperms353 sequences of *O. sativa* were downloaded from Kew Tree of Life Explorer and corrected by the CDS to serve as the gold standard for comparison with the results of Easy353 and HybPiper. All test files were deposited in Zenodo (https://doi.org/10.5281/zenodo.7350475). Based on the benchmark, we ran a comprehensive examination of the availability of the sequences, the rates of base-calling error and indel error of the sequences, between Easy353 and HybPiper (supplementarytable S1, Supplementary Material online). As the sequencing depth increased, the number and total length of available sequences recovered by Easy353 and HybPiper continuously grew, while the base-calling error rate and indel error rate decreased (fig. 1*E*–*G*). When the sequencing depth was <10×, deeper sequencing depths substantially increased the quantity and quality of inferred sequences. It is worth noting that the effect of indel errors on phylogeny could be reduced or eliminated by trimming the sequence alignment with software such as trimAl v1.4.15 (Capella-Gutiérrez et al. 2009). However, base-calling errors may result in the misestimation of branch lengths and topology of the phylogenetic tree, which can be hard to be eliminated. In all tests, the base-calling error rate of sequences recovered by Easy353 was <1% (0.10–0.65%), suggesting that the AGS recovered by Easy353 can be applied to phylogenetic research with high confidence.

Then, we used genome skimming and transcriptome sequencing data of five species (*Aegopodium podagraria*, *Anthriscus sylvestris*, *Cyclospermum leptophyllum*, *Foeniculum vulgare*, and *Haplosphaera phaea*) from our previous studies (Wen et al. 2020, 2021) and the target enrichment data from the Kew Tree of Life Explorer to validate the number and length of genes that can be recovered from different sequencing data. HybPiper was previously assessed (Johnson et al. 2019) based on the value of *T50*, a statistic that represents the number of genes for which the length of coding sequences recovered was at least 50% of the target length (the average length of the target instances for each gene). Similarly, we adopted the *T50* statistic to assess Easy353. In our experiments, both Easy353 and HybPiper had a *T50* of over 300 when using transcriptome and target enrichment sequencing data. However, Easy353 had a larger *T50*. For genome skimming sequencing data, Easy353 had a median *T50* of 298, whereas HybPiper had a median *T50* value of 27 (supplementary table S3, Supplementary Material online). These results indicate that Easy353 performs well with the AGS when using genome skimming, transcriptome, and target enrichment sequencing data. Based on our analyes and previous studies (Desai et al. 2013; Hou et al. 2013), we recommend using ∼20× coverage of the genome of target species as the most cost-effective strategy with Easy353.

In summary, Easy353 optimizes the read mapping and assembly algorithms and combines the position information on reference sequences to aid assembly. Compared with other pipelines, Easy353 can provide substantial benefits in recovering the AGS, including lowering sequencing costs, reducing assembly time, increasing AGS acquisition rate, and minimizing erroneous sequence recovery. Easy353 is user-friendly with simple settings and has accompanying tutorials that are freely available via GitHub (https://github.com/plant720/Easy353). Easy353 is licensed under the terms of the MIT license.

## Supplementary Material

msac261_Supplementary_Data
