# Supplementary material for: Easy353: A Tool to Get Angiosperms353 Genes for Phylogenomic Research
Source: Mol Biol Evol. 2022 Dec 2;39(12):msac261. doi: 10.1093/molbev/msac261 (PMC9757696; doi:10.1093/molbev/msac261)
Supplement: msac261_Supplementary_Data [file msac261_supplementary_data.zip › Supplementary Information.docx]

**Supplementary Information**

In this supplementary material, we provide further details of Easy353, including the detailed description of the algorithm of Easy353 and a side-by-side comparison on the methodology and outcomes between Easy353 and HybPiper

1. **The algorithm of Easy353**


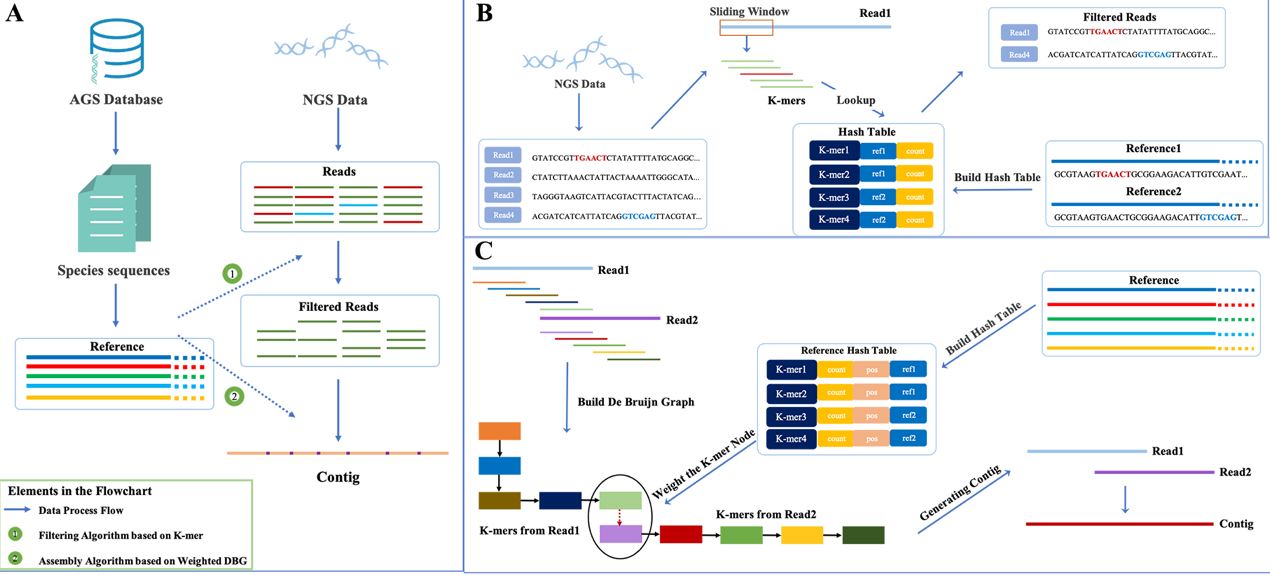


The workflow of Easy353 consists of three major steps: 1) Reference database building. Build a reference database with the user-defined taxa. 2) Read filtering. Use the partial fragment consistency between reference sequences and reads to classify the raw reads into individual reads of each gene locus. The reads related to the same gene locus are stored in the same individual file. 3) Read assembly. Assemble the reads related to each gene locus under the guidance of reference. Prior to running Easy353, users need to generate genomic reads for the target species of interest. The reads can be generated by high-throughput sequencing technology (including genome skimming, RNA-seq, and target enrichment).

- 1. **Reference database building**

Easy353 will automatically download available AGS sequences from the Kew Tree of Life Explorer at https://treeoflife.kew.org (Baker, et al. 2021) according to the user-defined organisms. The database would consist of the genus/family data to which the target species belongs. Subsequently, the downloaded AGS sequences would be stored in FASTA format as the reference.

- 1. **Read filtering**

Easy353 will filter out the reads related to AGS from sequencing data. In this step, all reference sequences were broken into k-mers and represented in a hash table. Then, each read was divided into k-mers and checked against the hash table for identical k-mers; if at least one identical k-mer was detected, the read was considered as a useful read for matching to the reference gene. We modified a read-mapping algorithm based on hash tables (Schbath, et al. 2012).

- 1. **Read assembly**

Easy353 assembles the reads related to each gene locus under the guidance of reference. The reads and reference sequences related to the same gene would be divided into k-mers, which would be stored in the read hash table and reference hash table, respectively. In the reference hash table, the key was k-mer, and the value was the count of k-mer; while in the read hash table, the key was k-mer, and the value was the count of k-mer and the pos of this k-mer (pos is the average percentage distance of k-mer on the reference). Then, the k-mers in the read hash table were used to build the de Bruijn graph (DBG) (Compeau, et al. 2011). In the DBG, each node represented a k-mer and there would be a directed edge between two nodes if an overlap with k-1 bases was detected. After building the DBG, the k-mers shared by reads and reference sequences would be used as seeds, which were the candidate nodes to start assembly. Among all the seeds, Easy353 would choose the one with the most occurrences as the initial node and a node with k-1 overlap with the initial node as the next node. When several successor nodes were identified, information of the reference sequences was used to weigh the nodes, and the subsequent node with the highest weights was selected as the next visited. The weight of the subsequent node was calculated as $W_{node}={count}^{\left( 1-position \right)}$, where the count was the abundance of current node, and the position is the distance difference between the current node and the previous node. Finally, the path with the most connected nodes in the DBG was selected as the assembly result.

1. **Comparison between Easy353 and HybPiper**

To demonstrate the value of Easy353, we provided a side-by-side comparison on the methodology and outcomes (data matrices and phylogenetic outcomes based on sequences from empirical studies) between Easy353 and HybPiper.

- 1. **Methodology comparison**

The HybPiper pipeline v2.0 (Johnson, et al. 2016) was used to recover both coding sequences (CDS) and their flanking intron/noncoding regions. The process includes three major steps: using the nuclear sequences of Angiosperms353 genes (Johnson, et al. 2019) as the references to capture all the reads from sequenced accessions via the BWA option with default seed length k =19 (Li and Durbin 2009), applying the SPAdes (Bankevich, et al. 2012) to assemble reads into long contigs, and implementing the intronerate.py module to recover “intron” and “supercontig” (CDS +intron fragments) sequences

Easy353 is a reference-guided gene assembler. It can capture the Angiosperms353 gene set (AGS) from transcriptome, the whole genome, and even genome skimming raw sequencing data with high accuracy and precision (See details above). Easy353 optimizes the hash table based read mapping (Schbath, et al. 2012) and de Bruijn graph based assembly algorithm (Compeau, et al. 2011; Bao, et al. 2014). It also accounts for the position information on reference sequences to aid assembly. Compared to HybPiper, Easy353 is an assembler with optimized algorithms and no dependencies, which can recover longer and more accurate sequences.

- 1. **Phylogenetic analysis**

We used nine transcriptomes from seven species of three major lineages of *Cornus* and outgroups (*Alangium chinense*, *Cornus alternifolia*, *Cornus capitata* subsp. *emeiensis*, *Cornus controversa*, *Cornus elliptica*, *Cornus florida*, *Cornus kousa*, *Cornus officinalis*, *Dichroa febrifuga*) to do the phylogenetic analyses.

- - 1. **Data Generation**

Raw transcriptome data were obtained from our previous research (Yu, et al. 2017). Sequences of the outgroups, *Alangium chinense* and *Dichroa febrifuga*, were generated by Beijing Genomics Institute (BGI) with 90 bp paired end sequencing on Illumina HiSeq 2000 and made available courtesy of the One Thousand Plants (1KP) Transcriptome Project (http://www.onekp.com). Transcriptome data for seven *Cornus* species were generated for this study at North Carolina State University (NCSU Genomic Science Lab) and BGI. For *Cornus kousa* and *C. elliptica*, normalized libraries were made using a TruSeq RNA library prep with mean size of 366 bp and 362 bp, respectively, and run for 72- bp paired-end sequencing on a GAIIx Illumina sequencer at the Genomic Science Lab at NCSU. For *Cornus alternifolia*, *C. capitata*, *C. controversa*, *C. florida*, and *C. officinalis*, non-normalized libraries were run for 150 bp paired-end sequencing on the Illumina HiSeq-2000 platform.

- - 1. **Locus Data Assembly and MSA Generation**

The HybPiper pipeline v2.0 (Johnson, et al. 2016) was used to recover both coding sequences (CDS) and their flanking intron/noncoding regions with default parameters. Due to the low coverages of raw sequencing reads, the certain parameters were set in Easy353 to recover supercontig sequences, including filter_kmer = 21, assemble_kmer = 31, and kmer_limit = 4. The major steps of Easy353 and HybPiper were following the aforementioned description.

We used a homemade script to extract the recovered genes for each species and saved the same locus from different species in a single FASTA file. We only kept the locus recovered among all species for analysis. Next, we used MUSCLE v3.8.31 (Edgar 2004) to generate multiple sequence alignment and used TrimAl v1.4.15 (Capella-Gutiérrez, et al. 2009) to remove spurious sequences or poorly aligned regions via the default options.

- - 1. **Coalescent-based species tree**

We employed the RaxML v8.2.12 (Stamatakis 2014) to generate gene trees and conducted ASTRAL-III (Zhang, et al. 2018) to generate the coalescent-based species trees. All gene trees from RaxML for both genera were set as the input for ASTRAL-III with the default parameters.

The recovered sequences, data matrices, and phylogenetic trees were deposited at <https://github.com/plant720/Easy353Test>. Based on the comparison results, we revealed that Easy353 could recover more genes with longer lengths with more phylogenetic information. The phylogenetic trees build from recovered sequences had the same topology, indicating they can be used for downstream phylogenetic analysis.

**Reference**

Baker WJ, Bailey P, Barber V, Barker A, Bellot S, Bishop D, Botigué LR, Brewer G, Carruthers T, Clarkson JJ, et al. 2021. A Comprehensive Phylogenomic Platform for Exploring the Angiosperm Tree of Life. *Syst Biol.* 71:301-319.

Bankevich A, Nurk S, Antipov D, Gurevich AA, Dvorkin M, Kulikov AS, Lesin VM, Nikolenko SI, Pham S, Prjibelski AD, et al. 2012. SPAdes: a new genome assembly algorithm and its applications to single-cell sequencing. *J Comput Biol.* 19:455-477.

Bao E, Jiang T, Girke T. 2014. AlignGraph: algorithm for secondary de novo genome assembly guided by closely related references. *Bioinformatics.* 30:i319-i328.

Capella-Gutiérrez S, Silla-Martínez JM, Gabaldón T. 2009. trimAl: a tool for automated alignment trimming in large-scale phylogenetic analyses. *Bioinformatics.* 25:1972-1973.

Compeau PEC, Pevzner PA, Tesler G. 2011. How to apply de Bruijn graphs to genome assembly. *Nat Biotechnol.* 29:987-991.

Edgar RC. 2004. MUSCLE: multiple sequence alignment with high accuracy and high throughput. *Nucleic Acids Res.* 32:1792-1797.

Johnson MG, Gardner EM, Liu Y, Medina R, Goffinet B, Shaw AJ, Zerega NJC, Wickett NJ. 2016. HybPiper: Extracting coding sequence and introns for phylogenetics from high-throughput sequencing reads using target enrichment. *Appl Plant Sci.* 4:apps.1600016.

Johnson MG, Pokorny L, Dodsworth S, Botigue LR, Cowan RS, Devault A, Eiserhardt WL, Epitawalage N, Forest F, Kim JT. 2019. A universal probe set for targeted sequencing of 353 nuclear genes from any flowering plant designed using k-medoids clustering. *Syst Biol.* 68:594-606.

Li H, Durbin R. 2009. Fast and accurate short read alignment with Burrows-Wheeler transform. *Bioinformatics.* 25:1754-1760.

Schbath S, Martin V, Zytnicki M, Fayolle J, Loux V, Gibrat JF. 2012. Mapping reads on a genomic sequence: an algorithmic overview and a practical comparative analysis. *J Comput Biol.* 19:796-813.

Schneeberger K, Ossowski S, Ott F, Klein JD, Wang X, Lanz C, Smith LM, Cao J, Fitz J, Warthmann N. 2011. Reference-guided assembly of four diverse Arabidopsis thaliana genomes. *Proceedings of the National Academy of Sciences.* 108:10249-10254.

Schneeberger K, Ossowski S, Ott F, Klein JD, Wang X, Lanz C, Smith LM, Cao J, Fitz J, Warthmann N, et al. 2011. Reference-guided assembly of four diverse Arabidopsis thaliana genomes. *Proc Natl Acad Sci U S A.* 108:10249-10254.

Stamatakis A. 2014. RAxML version 8: a tool for phylogenetic analysis and post-analysis of large phylogenies. *Bioinformatics.* 30:1312-1313.

Yu Y, Xiang Q, Manos PS, Soltis DE, Soltis PS, Song B-H, Cheng S, Liu X, Wong G. 2017. Whole-genome duplication and molecular evolution in Cornus L. (Cornaceae) – Insights from transcriptome sequences. *PLoS ONE.* 12:e0171361.

Zhang C, Rabiee M, Sayyari E, Mirarab S. 2018. ASTRAL-III: polynomial time species tree reconstruction from partially resolved gene trees. *BMC Bioinformatics.* 19:15-30.
