# Supplementary material for: Easy353: A Tool to Get Angiosperms353 Genes for Phylogenomic Research
Source: Mol Biol Evol. 2022 Dec 2;39(12):msac261. doi: 10.1093/molbev/msac261 (PMC9757696; doi:10.1093/molbev/msac261)
Supplement: msac261_Supplementary_Data [file msac261_supplementary_data.zip › Table S1.docx]

|  |  | level1 | level2 | level3 | level4 | total_seq | base-calling error | indel | base_rate | indel_rate | total_len |
| --- | --- | --- | --- | --- | --- | --- | --- | --- | --- | --- | --- |
| 1x | Easy353 | 6 | 34 | 1 | 14 | 55 | 144 | 204 | 0.56% | 0.79% | 25873 |
|  | HybPiper | 0 | 0 | 0 | 0 | 0 | 0 | 0 | N/A | N/A | 0 |
| 5x | Easy353 | 94 | 124 | 1 | 37 | 256 | 280 | 166 | 0.15% | 0.09% | 187705 |
|  | HybPiper | 20 | 63 | 11 | 29 | 123 | 785 | 3224 | 0.87% | 3.56% | 90443 |
| 10x | Easy353 | 157 | 132 | 0 | 36 | 325 | 552 | 120 | 0.21% | 0.04% | 267506 |
|  | HybPiper | 54 | 166 | 16 | 69 | 305 | 706 | 2703 | 0.31% | 1.20% | 225144 |
| 20x | Easy353 | 216 | 99 | 0 | 19 | 334 | 491 | 99 | 0.17% | 0.03% | 292947 |
|  | HybPiper | 63 | 203 | 19 | 53 | 338 | 827 | 3104 | 0.34% | 1.28% | 241972 |
| 50x | Easy353 | 254 | 69 | 0 | 12 | 335 | 348 | 110 | 0.11% | 0.04% | 307088 |
|  | HybPiper | 74 | 197 | 17 | 50 | 338 | 542 | 2235 | 0.22% | 0.92% | 241972 |

**Comparison of the accuracy of Easy353 and HybPiper**

All of the parameters used are the defaults. The default parameters used by Easy353 are *kmer_limit* =8 and *k2*=41.

*Kmer_limit* is the limit of the *k*-mer count. The *kmer_limit* is used to remove erroneous, low-abundance *k*-mers. This parameter strongly depends on the dataset. It corresponds to the smallest amount of times a correct *k*-mer appears in the reads. A value is 8, which means only *k*-mers with at least 9 occurrences will be used for assembly.

*k2* is the k-mer length setting for assembly. The *k2* is the length of the nodes in the de Bruijn graph (DBG). In read assembly, the filtered reads are divided into *k*-mers; the *k*-mers are employed as the nodes of the DBG. It also strongly depends on the input dataset.

depth: the coverage of simulated data for the CDS of Oryza sativa

level1: the gene for which the coding sequence recovered was 100% identity with the gold standard and 100% coverage

level1: the gene for which the coding sequence recovered was 100% identity with the gold standard and 100% coverage

level2: the gene for which the coding sequence recovered was 99~100% identity with the gold standard and 50~100% coverage

level3: the gene for which the coding sequence recovered was 99~100% identity with the gold standard and 0~50% coverage

level4: the recovered gene that was not attributed to level1-level3

total_seq: the number of all recovered sequences

base-calling error: the total number of all base-calling errors on the recovered sequences

indel: the total number of all indel errors on the recovered sequences

base_rate: the base-calling error rate, which is the rate of base-calling error sites in the recovered sequences to the total bases

indel_rate: the indel error rate, which is the rate of indel sites in the recovered sequences to the total bases

total_len: the total number of bases on the recovered sequence
